# Health utilities for chronic low back pain

**DOI:** 10.1186/s12995-017-0172-7

**Published:** 2017-09-02

**Authors:** Anna Lene Seidler, Constanze Rethberg, Jochen Schmitt, Albert Nienhaus, Andreas Seidler

**Affiliations:** 1 Institute and Policlinic of Occupational and Social Medicine (IPAS), Faculty of Medicine, Technische Universität Dresden, Dresden, Germany; 2 NHMRC Clinical Trials Centre, Systematic Reviews & Health Technology Assessment, University of Sydney, Sydney, Australia; 3 Center for Evidence-Based Healthcare, University Hospital and Medical Faculty Carl Gustav Carus, TU Dresden, Dresden, Germany; 4 German Social Accident Insurance Institution for the Health and Welfare Service, Hamburg, Germany; 5 Institute for Health Services Research in Dermatology and Nursing, University Medical Center Hamburg-Eppendorf, Hamburg, Germany

**Keywords:** Chronic Low back pain, Health utilities, Health economics, Time-trade-off

## Abstract

**Background:**

Chronic low back pain (LBP) is a common health problem, with a large potential for primary prevention. Health utilities (HU) reflect which proportion of their expected remaining life time individuals would hypothetically trade to be alleviated of a health condition of interest. A value of 0 means “prefer to die immediately”, a value of 1 means “not willing to trade any life time”. The aim of this cross-sectional study was to assess HU for LBP patients and for healthy participants and to examine whether HU for LBP are useful indicators to substantiate preventive and therapeutic decision making.

**Methods:**

Healthy participants (*n* = 126) and LBP patients (*n* = 32) were recruited mainly among the employees of a tertiary care hospital in Germany. Standardized LBP scenarios were presented to all participants and HU values were assessed using the time-trade-off method.

**Results:**

Median HU for LBP were 0.90 (IQR 0.31) for participants and 0.93 (IQR 0.10) for LBP patients. Measurements were consistent across illness severity ratings with HU and with a visual analogue scale (VAS); in the healthy sample the intraclass correlation coefficient (ICC) was 0.61 (95% CI 0.23–1.00, F(1125) = 190, *p* < .001), in the patient sample the ICC was 0.66 (95% CI = 0.24–1.00, F(1,31) = 62, *p* < .001). 8% of participants reported HU of 1. There was no statistically significant relation between HU and age, income, or gender.

**Conclusion:**

On average, participants chose a 7 to 10% shorter life expectancy to avoid LBP, but almost 1 in 10 participants were not willing to trade any life years. The results indicate a certain stability of HU due to the comparability of HU ratings across patients and healthy participants, the measurement consistency when comparing VAS and HU ratings, and the lack of association between demographic variables and HU. This underlines the usefulness of HU for measuring illness severity in comparative health economics evaluations of preventive and therapeutic measures that address chronic LBP or other pain-characterized diseases. Future studies should focus on different LBP intensities and derive stratified HU that reflect the distribution of pain intensity in the population.

## Background

Chronic low back pain (LBP) is a common health problem. The global 1-month prevalence of LBP has been estimated to be 23.2 ± 2.9% [1]. Assessing and addressing chronic LBP – commonly defined as LBP of more than 12 weeks duration [2] – is highly relevant not only to general medicine and orthopedics, but also to occupational medicine [3, 4]. Chronic LBP is a leading cause of work absence worldwide which puts a massive economic burden on both the society and individuals [5–7]. It is related to the working conditions in many different job sectors, since it is associated with physically straining work-tasks (e.g. heavy lifting, forward bending, whole-body vibration) and psychosocial work factors (e.g. high demand and low control) [8]. A review of occupational LBP and preventive approaches found that there is high potential for work-related primary prevention of chronic LBP [9]. Decision making concerning occupational prevention measures and treatments for chronic LBP requires health economic evaluations and cost-utility analyses. A systematic review of previous cost utility studies for LBP identified a need for additional studies [10].

Health utilities (HU) are a measure of illness severity that are widely used to facilitate cost-effectiveness analyses and health-related policy making [11]. HU assessed with the time-trade-off method provide a measure of health-related quality of life based on decision-making theory: a value of 0 would mean “prefer to die immediately”, a value of 1 would mean “not willing to trade any life time”. HU scores reflect which proportion of their life time affected patients and non-affected healthy individuals would hypothetically trade to be alleviated of a respective condition [12]. For instance, a score of 0.6 indicates that an individual would choose to live 60% of their (hypothetical) remaining life time in perfect health over living their full remaining life time with the respective condition. HU can be used to assess trade-offs relevant for resource allocation [13] and they allow for a direct comparison of disease outcomes across different health conditions [14]. HU for chronic LBP could provide a tool to conduct cost-utility analyses for treatments and occupational prevention measures and to compare the cost-utility of occupational health promotion targeting chronic LBP to occupational health promotion addressing different diseases such as mental illnesses [15–17].

A previous study assessing patient utilities for LBP found an average utility of 0.93 for mild, 0.65 for moderate, and 0.18 for severe back pain [18]. However, the sample was highly selective; it consisted of 41 LBP patients in a tertiary care hospital. The derived HU are likely to not represent generalizable views on chronic LBP and might thus not be suitable for work-related as well as lifestyle-related primary preventive interventions for healthy individuals. Due to the high potential of occupational preventative approaches targeting healthy individuals, it is important to assess health utility values in this healthy target group. Another previous study examined HU in a healthy sample for chronic pain in general but not chronic LBP specifically [19]. The authors found mean HU of 0.84 for mild, 0.72 for moderate, 0.04 for severe and −0.15 for very severe chronic pain; the negative value indicates that participants would prefer death to suffering these pain. However, the usefulness of HU varies across disease type [20]. While the cited study gives important information on healthy individuals’ ratings of chronic pain and shows that the HU are sensitive to severity of pain, it remains unclear whether these general values are transferable to chronic LBP.

The primary aim of the current study was to assess HU for chronic LBP in a sample of LBP patients and a healthy sample. The secondary aim was to examine whether HU for chronic LBP are useful indicators to substantiate decision making generally and in preventive (e.g. occupational) medicine specifically by assessing: how HU compare to a standard measure of pain severity (VAS-scale) and other severity measures; whether there are many individuals not willing to trade any life years; and whether HU derived with the time-trade-off method are influenced by other variables (age, gender, and own level of pain).

## Methods

### Participants

The data for this cross-sectional study were collected between October 2011 and August 2015 at the Institute and Policlinic of Occupational and Social Medicine and the Center for Evidence-Based Healthcare, Faculty of Medicine of the TU Dresden. The study population included two groups of participants: 126 healthy participants and 32 patients with chronic LBP. Eligible for inclusion were only participants that: were at least 18 years old; were affiliated with University Hospital and Faculty of Medicine Dresden; and that gave informed consent to participate in the study. Exclusion criteria were: lack of willingness to co-operate and lack of understanding of the study questions. Individuals were eligible for inclusion as patients if an ICD-10 code M54.5 was specified by a careful medical examination and anamnesis and if they reported a LBP duration of more than 3 months, which is in accordance with a widely accepted definition of chronic LBP [2, 9]. Patients with specific lumbar spine diseases (e.g. disc herniation, lumbago with sciatica) were excluded. The healthy participants were working in health-related sectors, to ensure the ability to make informed judgements about LBP. The healthy sample was considered the primary study population to derive HU for healthy individuals as recommended for health economic analyses [17]. The patient group was interviewed to specifically assess the influence of being personally affected by chronic LBP on HU.

The sample was recruited from the employees at the University Hospital and Faculty of Medicine Dresden, and through the social media presence and the intranet of the University Hospital and Faculty of Medicine. The study protocol was approved by the responsible ethics committee of the University Clinic Dresden. This manuscript follows the STROBE Statement for cross-sectional studies [21].

### Data collection and measures

Data were collected through a standardized computer-assisted interview. Firstly, participants’ age, sex, and income group were assessed. For employees of the University Hospital no direct information on income was available, their income was thus estimated based on their occupational role. After assessing this demographic information, a standardized scenario for chronic LBP was presented to all participants to assess HU using the time-trade-off method (see Table 1). Participants were given the choice between living for their remaining life expectancy (calculated by the software based on participants’ age and sex) with the described chronic LBP or for a shorter duration in perfect health. The shorter duration in perfect health was a randomly chosen duration based on a normal distribution with mean (± SD) = participant’s remaining life expectancy × 0.8 (±0.05) to avoid a starting point bias [22]. The duration of life in perfect health was then altered until the individual’s preferences were equal. HU were calculated by taking the ratio between the duration of life in perfect health and the duration of life with chronic LBP. Additionally, participants were asked to rate the severity of the standardized LBP scenario on a visual analogue scale (VAS) with 0 reflecting perfect health and 100 reflecting worst imaginable condition. Willingness to pay (WTP) was assessed by asking participants how much they would be willing to pay monthly to not suffer from chronic LBP, and how much they think health insurances should spend monthly so people do not have to suffer from chronic LBP.

**Table 1:** Health state scenario

	Unspecified chronic low back pain
Disease Duration	>3 months
Affected Regions	Low back, one-or-two sided, can radiated to other body regions
Pain Sensations	Pain, sensory disturbances, sometimes numbness and tingling sensations in the legs
Causes/Detoriation	Overstraining, lack of exercise, relieving postures, psychosocial factors, being overweight, smoking
Treatment	Symptom-oriented: Physical activity (e.g. physiotherapy), possibly analgesics
Impact on Life	Occupational impacts, limitations everyday activities and interpersonal relationships

The patient group suffering from chronic LBP rated their current pain with the time-trade-off method and on a VAS scale; their level of disability was assessed using the Roland Morris disability index [23]. Impairment and productivity were assessed using the Work Productivity and Activity Impairment (WPAI) questionnaire [24]. Furthermore, patients completed the Center for Epidemiological Studies Depression Scale (CES-D) [25] to measure depressive symptoms that may systematically influence HU ratings [26].

We conducted a sample size calculation assuming a standard deviation of 0.1 for the patient sample and of 0.2 for the healthy sample. A sample size of 32 LBP patients and 126 healthy individuals was necessary to obtain 95% confidence intervals with a width of 0.1.

### Statistical analysis

Descriptive statistics were calculated for all variables. Arithmetic means with standard deviations, and medians with interquartile range were calculated to derive average disease severity values for HU, VAS and WTP. The key variables were not normally distributed. Thus, robust Spearman correlation analysis and robust regression analysis were applied to assess relations between the disease severity measures and third variables. Measurement consistency between HU and VAS values was tested by calculating two-way random effect intraclass correlation coefficients (ICCs). T-tests were performed to assess group differences. Welsh t-tests were applied when Levene Tests revealed that the variances were not homogenous. All analyses were performed using the open-source software R [27].

## Results

Sociodemographic and disease specific characteristics of study participants are shown in Table 2. There were no missing data for any individuals or variables. The mean age of patients with chronic LBP was 41 years (SD = 13), they were thus considerably older than the group of healthy participants (mean age: 30 years, SD = 9). Furthermore, a slightly higher percentage of LBP patients were in the lower income groups compared to the healthy participants (see Table 2). Chronic LBP was a well-known disease in the group of healthy participants (that were mainly health professionals) not suffering from LBP: The vast majority (94%) had a good idea of what chronic LBP is, and 62% had friends or relatives suffering from LBP.

**Table 2:** Sociodemographics and disease-specific characteristics of study participants

Variable	Healthy participants *n* = 126	Patients with chronic low back pain *n* = 32
Sex (% female)	54%	59%
Age in years (mean, SD)	29.95 (9.25)	41.03 (12.85)
Income group (%)		
<1000 €/month	10%	16%
1000–2000 €/month	2%	13%
2000–3000 €/month	56%	48%
3000–4000 €/month	30%	19%
>4000 €/month	2%	3%
Having idea of what low back pain is (%)	94%	100%
Friends/family members with low back pain (%)	62%	66%
Family history of LBP (positive, %)	-	56%
Disease duration in years (mean, SD)	-	15.48 (9.48)
Roland Morris disability index		
light (%)	-	72%
moderate (%)	-	25%
severe (%)	-	3%
Degree health affected productivity while working (0 to 1) (WPAI) (mean, SD)	-	0.18 (0.18)
Degree health affected regular activities (0 to 1) (WPAI) (mean, SD)	-	0.28 (0.24)
Depressive Symptoms (CES-D) (mean, SD)	-	12.06 (7.52)
HU own disease (0 to 1) (mean, SD)	-	0.93 (0.13)
Pain own disease (100 mm VAS) (mean, SD)	-	22.25 (21.60)

*SD* Standard Deviation, *WPAI* Work Productivity and Activity Impairment Questionnaire, *CES-D* Center for Epidemiological Studies Depression Scale, *VAS* Visual Analogue Scale, − not applicable

For the group suffering from chronic LBP, the mean disease duration was 15.48 (SD = 9.48; range 1–40) years. According to the Roland Morris Disability index, the degree of disability was light for 72% (*n* = 23), moderate for 25% (*n* = 8), and severe for 3% (*n* = 1) of the patients. This relatively light impairment for most LBP patients is also reflected in the HU for their current pain assessed with the time-trade-off method (median = 0.96, IQR = 0.06), and in the ratings of current pain on a VAS scale (mean = 22.25, SD = 21.60). The mean VAS of LBP patients with a light degree of disability (according to the Roland Morris Disability index) was 14.78 (median 11.0), the mean VAS of patients with a moderate degree of disability was 39.75 (median 32.5), the only patient with a severe degree of disability had a VAS of 54.

### HU values for healthy participants and LBP patients

**Fig. 1:**
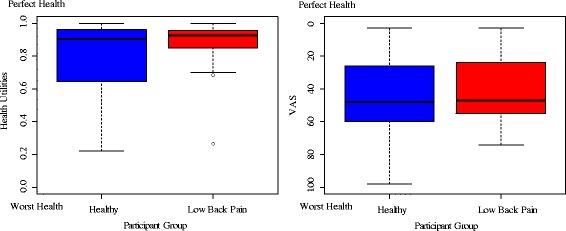
Median and interquartile range of Health Utilities and VAS values by participant group

Median HU for the standardized chronic LBP scenario were 0.90 (*IQR* = 0.31) as rated by healthy participants and 0.93 (*IQR* = 0.10) as rated by LBP patients. They were thus comparable between the groups. The mean difference between healthy individuals (*M* = 0.81, *SD* = 0.19) and LBP patients (*M* = 0.88, *SD* = 0.14) was statistically significant (*t*(63.64*)* = −2.48, *p* = 0.02). However, the less pronounced median difference indicates that the mean difference is related to more extreme ratings in the healthy group (see Table 3 and Fig. 1).

**Table 3:** HU and VAS values for standardized chronic low back pain scenario for different groups

Group	HU		VAS		WTP own money		WTP health insurance	
	Mean (SD)	Median (IQR)	Mean (SD)	Median (IQR)	Mean (SD)	Median (IQR)	Mean (SD)	Median (IQR)
Healthy (*n* = 126)	0.81 (0.19)	0.90 (0.31)	44.34 (20.84)	48.00 (34.00)	236.03 (314.98)	150.00 (200.00)	664.93 (945.21)	300.00 (500.00)
Low Back Pain (*n* = 32)	0.88 (0.14)	0.93 (0.10)	40.91 (19.64)	47.00 (30.50)	180.78 (269.78)	100.00 (150.00)	785.31 (1234.48)	300.00 (410.00)
Total (*n* = 156)	0.83 (0.18)	0.91 (0.25)	43.65 (20.59)	47.00 (33.75)	224.84 (306.36)	150.00 (170.00)	689.94 (1008.83)	300.00 (528.75)

*HU* Health Utilities, *VAS* 100 mm Visual Analogue Scale Values, *WTP* Willingness to Pay Monthly in Euros, *SD* Standard Deviation, *IQR* Interquartile Range

### Relation of HU to VAS and other severity measures

Mean, standard deviation, median and interquartile range for the HU, VAS, and WTP scores for the standardized scenarios are shown in Table 3. There was a good reliability across VAS and HU, the two-way random effects ICC was .61 (95% CI = 0.23–1.00, F(1125) = 190, *p* < .001) for the healthy sample, and .66 (95% CI = 0.24–1.00, F(1,31) = 62, *p* < .001) for the patient sample. Correlations between the different measures of rated illness severity are shown in Table 4. HU and VAS are moderately correlated, interestingly, the correlation between HU and VAS is stronger for the group of healthy participants (*r* = −.53, *p* < .001) than for the patient group (*r* = −.31, *p* = .09). VAS and HU show a stronger correlation and thus seem to be more related constructs than VAS and WTP or HU and WTP.

**Table 4:** Spearman correlation coefficients for different measures of illness severity for different groups

Group	HU ↔ VAS	HU ↔ Own WTP	HU ↔ Insurance WTP	VAS ↔ Own WTP	VAS ↔ Insurance WTP	Own WTP ↔ Insurance WTP
Healthy (*n* = 126)	−.53***	−.29***	−.15	.22*	.20*	.67***
Low Back Pain (*n* = 32)	−.31+	−.20	−.10	−.11	−.04	.73***
Total (*n* = 156)	−.50***	−.30***	−.15+	.18*	.16*	.68***

*HU* Health Utilities, *VAS* 100 mm Visual Analogue Scale Values, *WTP* Willingness to Pay, + = *p* < .01, * = *p* < .05, ** = *p* < .01, *** = *p* < .001

### Individuals not willing to trade any life years

As shown in Fig. 1, ten of the healthy participants (8%), were not willing to hypothetically trade any life years to be relieved of the presented standardized scenario of chronic LBP (and thus received HU of 1). In comparison, no healthy participant rated chronic LBP as equal to perfect health on a VAS scale, and only one healthy participant was not willing to pay any money to be relieved of LBP. Similarly, three of the LBP patients (9%) had HU values of 1, but no LBP patient rated the disease as equal to perfect health on a VAS scale, and no patient was not willing to pay any money to be relieved of LBP.

### Demographic factors, own pain, and HU

**Fig. 2:**
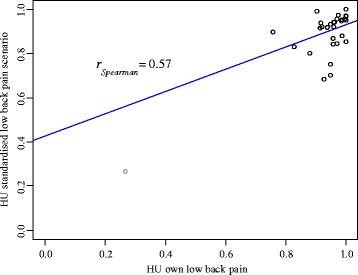
Association between Health Utilities rating of own LBP and Health Utilities rating of standardized LBP scenario

In the sample, younger people were willing to trade a higher proportion of life years (since they reported lower HU), however, this association was not statistically significant (*r*(156) = .10, *p* = .24). VAS and age do not seem to be related in the sample, if anything, younger people rated chronic LBP as less severe (*r* = .05, *p* = .55). Males were willing to trade slightly more life years (*M*_*male*_ = 0.81, *SD*_*male*_ = 0.19, *M*_*female*_ = 0.83, *SD*_*female*_ = 0.18), but again this difference was not statistically significant (*t*(156) = −0.67, *p* = .50). There was no sex difference for VAS (*M*_*male*_ = 44.24, *SD*_*male*_ = 21.38, *M*_*female*_ = 43.16, *SD*_*female*_ = 20.02, *t*(156) = 0.33, *p* = .74). There was a tendency (albeit not statistically significant) for individuals in the higher income group to report higher HU (*r* = .12, *p* = .14), while income group did not influence VAS ratings (*r* = −.01, *p* = .92). As shown in Fig. 2, there was a positive relation for chronic LBP patients between HU for their own LBP, and HU for the standardized description of LBP (*r* = .57, *p* < .001).

## Discussion

This study derived HU values with the time-trade-off method for a sample of patients with chronic LBP and a sample of healthy participants. Median HU for chronic LBP in the healthy sample were 0.90, while median HU in the LBP patient sample were 0.93. HU derived with the time-trade-off method were related to other severity measures and particularly VAS, and there was good measurement consistency when comparing HU and VAS. This indicates that HU for chronic LBP reflect a fairly direct severity rating of LBP and are thus a useful indicator of illness severity. The usefulness of HU is also supported by the lack of statistically significant associations between HU and demographic variables (age, gender, and income). However, almost one in ten participants (in both the healthy and the patient sample) was not willing to trade any life years to be relieved of chronic LBP, while no participant rated chronic LBP as equivalent to being perfectly healthy on a VAS scale.

This observed ceiling effect that exists in HU derived with the time-trade-off method but not in other severity measures has previously been reported for other diseases but not for chronic LBP [20]. On the one hand, this effect impairs the interpretability of HU, as it is not possible to differentiate between ‘perfect health’ and ‘condition not worse enough to trade life years’. It also impairs the direct comparability between HU and other measures of disease severity [20]. On the other hand, the ceiling effect provides useful information for the planning of prevention and intervention measures: It indicates that measures against chronic LBP including the risk of a reduced life expectancy or of other adverse side effects would not be accepted by some individuals since some individuals would not trade any life years to not suffer LBP.

The assessed associations between HU and potential influencing factors (e.g. age or sex) were stronger than the assessed associations between VAS and these factors. While none of these associations were statistically significant, this may have been due to the small sample size, since previous studies did find significant associations: Dolan & Roberts [20] used the time-trade-off method to assess health preferences for different health states (but not LBP) and found that age, sex and marital status influenced the derived values. HU increased up to the age of 45 and then started to fall until the age of 70. Men, and married or cohabiting individuals showed higher HU compared to women and individuals living alone. Additionally, mental health states such as depression have previously been shown to influence HU for cancer patients [26]. It is likely, that mental health states also influence HU for chronic LBP, since depression changes people’s outlooks on life and death (which the extreme case of suicidality illustrates).

Generally, death is a very vague concept and may mean something different to each individual. The perception of life and death possibly largely influences whether an individual would be willing to trade life years and if so how many to be relieved from a disease. A reason for this may be that people with depression place a lower value on life, while it may not reflect perceived severity of the illness. This is related to the finding that there are inconsistencies in HU values that can be attributed to loss aversion, the idea that people are more sensitive to losses than to gains [28]. There is strong evidence showing a higher prevalence of depression among chronic LPB patients [29, 30], it is thus possible that co-morbid depression might also influence HU ratings. These findings pose two difficulties. Firstly, it is difficult to interpret whether differences between population groups result from differing evaluations of the respective health state or of differing evaluations of the value of a life year. For instance, a married or cohabiting person may live a happier life and thus be less likely to trade these happy life years, despite judging the respective health state similarly severe as a person living alone. Secondly, HU being influenced by individual characteristics makes them less informative for cost-effectiveness analyses that concern the general population since it reduces their generalizability.

The HU for chronic LBP found with the time-trade-off method in this study are comparable to the mean HU of 0.93 for mild LBP found in a previous study with a patient sample [18], and they are slightly higher than HU assessed in a healthy sample for general mild chronic pain that were on average 0.84 [19]. This indicates that the LBP scenario used in this study to describe chronic LBP was perceived as a fairly mild type of pain. This is in line with a study comparing the perceived severity of different illnesses, showing that LBP is perceived as comparably moderate [31].

A strength of this study is that HU were assessed for both healthy participants and patients with chronic LBP. Looking at those two different samples is particularly useful for this particular disease: chronic LBP is often addressed in preventive health promotion programs. Because healthy individuals are the target of these programs, it is their rating that is relevant to inform decision makers, while for therapeutic interventions patient ratings are more relevant. Furthermore, this design allows a comparison between these two groups allowing for a judgement on the influence of perspective on HU. There were no drop-outs or missing values for any of the core variables (HU, VAS, WTP, age, gender). The risk of interviewer bias was minimized since all the interviews were conducted by the same well-trained interviewer, and the interviews were computer-assisted to standardize the testing procedure. The concept of chronic LBP was well known to the vast majority of healthy participants, reducing the risk of arbitrary values.

A major limitation to this study is that the sample is not representative of the general population. The average Roland Morris disability index of the current sample was lower (5.34) than that of a population-representative sample of LBP patients in a Greek survey (average Roland Morris disability index 10.01) [32]. This principally impacts the generalizability of the results. However, the mentioned Greek study gives the mean Roland Morris disability index of all individuals that suffered from LBP at the time of the survey, whereas the present sample also includes patients with chronic LBP that are currently free of pain (or almost free of pain). If only looking at individuals in our sample that are currently suffering from LBP (defined as pain levels of at least 20 out of 100 on a VAS scale), the mean Roland Morris disability index of this subsample was 9.09, and is thus comparable to the population-representative Greek study. Moreover, we found similar values for healthy participants and patients, this indicates a certain stability of the derived HU.

Furthermore, the use of a restricted sample of mainly health professionals ensures that healthy participants were informed about chronic LBP. Pain is a hard concept to grasp for someone not suffering from it, since it is invisible and hard to describe. Health professionals are likely to have better insight to this illness since they encounter people suffering from it and are likely to have factual knowledge on chronic LBP and its implications. They are therefore likely to be able to make more informed judgements. The sample size was too small to precisely assess the influence of potential covariates on HU ratings. Future studies should use larger and population-based samples. There was an age difference of 11 years between healthy participants and patients which may have introduced systematic differences to the HU ratings of the two groups. However, this concern is slightly alleviated by the finding that there was no statistically significant association between age and HU ratings.

For health economic evaluations of (primarily) preventable disorders like chronic LBP a reflection of the disease expression in the general population is of particular importance. In the chronic LBP description used in this study, we did not give explicit indications of pain intensity. However, the description seems to reflect moderate pain intensity since the mean VAS as rated by all LBP patients for the standardized chronic LBP scenario was 40.91 which is comparable to the mean VAS of 39.75 that LBP patients with a moderate degree of disability attributed to their own complaints. The advantage of not giving explicit indications of pain intensity is that it reflects how the intensity of chronic LBP varies in the population. The disadvantage of this approach is that it impedes the concrete description of an illness that is primarily pain based. This problem becomes clear when comparing the patient and the population group in our sample: On average, healthy participants rated chronic LBP as more severe than patients with chronic LBP did (note that most chronic LBP patients in our sample reported only light pain intensities). Furthermore, patients with more severe LBP rated the standardized chronic LBP scenario as more severe than patients with less severe LBP (Fig. 2). Consequently, future studies should incorporate explicit descriptions of a range of different pain intensities in their chronic LBP scenarios and derive HU for different levels of pain intensity. HU could then be weighted with respect to the distribution of pain intensities in the general population to derive values that can be used for a health economic evaluation of primary prevention programs. Pain-intensity stratified HU could also be useful for the health economics evaluations of therapeutic treatments, since often chronic LBP cannot be cured completely, but it can be eased. Ideally, these stratified values would be supplemented by HU for a pain reduction from strong to moderate and from moderate to light to optimize the evaluation of curative measures. Overall, deriving HU stratified by pain intensity should be the next step to improve the applicability of HU values to evaluate preventive and pain reduction measures for chronic LBP.

## Conclusion

The present study shows that on average chronic LBP patients chose a 7% shorter life expectancy and healthy participants chose a 10% shorter life expectancy to avoid chronic LBP, but almost 1 in 10 participants were not willing to trade any life years. Since LBP is one of the most common diseases in the population and has high preventive potential, these HU can give decision makers in preventive health promotion and health care valuable insight to how patients and healthy individuals perceive chronic LBP. Furthermore, the results indicate a certain stability of HU due to the comparability of HU ratings across patients and healthy participants, the measurement consistency and association between VAS and HU, and the lack of association between demographic variables and HU. The results point towards an applicability of HU for assessing illness severity of LBP specifically and the applicability of HU for diseases that are mainly characterized through pain in general. Future studies looking at pain-characterised diseases should include explicit descriptions of pain intensities in their disease scenarios and independently assess HU for these different pain intensities ideally in a sample that is representative of the population of interest.

## Abbreviations

CES-D Scale: Center for Epidemiological Studies Depression Scale
HU: Health Utilities
ICC: Intraclass Correlation Coefficient
IQR: Interquartile Range
LBP: Low Back Pain
M_female_: Mean Among Females
M_male_: Mean Among Males
r: Correlation Coefficient
SD: Standard Deviation
VAS: Visual Analogue Scale
WPAI Scale: Work Productivity and Activity Impairment Scale
WTP: Willingness to Pay
